# A review of quality of life themes in Duchenne muscular dystrophy for patients and carers

**DOI:** 10.1186/s12955-018-1062-0

**Published:** 2018-12-19

**Authors:** Lesley Uttley, Jill Carlton, Helen Buckley Woods, John Brazier

**Affiliations:** School of Health and Related Research (ScHARR), The University of Sheffield, Regent Court, 30 Regent Street, Sheffield, S1 4DA UK

**Keywords:** Duchenne muscular dystrophy, Literature review, Quality of life, Thematic analysis, Wider family impact

## Abstract

Duchenne Muscular Dystrophy (DMD) is a severe, life-limiting and incurable condition. However, studies estimating quality of life and those measuring actual quality of life in people living with DMD vary considerably. This discrepancy indicates potential difficulties with assessing quality of life using common generic quality of life instruments in this rare and unique population. This study sought to document the range of themes relevant to quality of life for people with DMD by examining the published literature and additionally to investigate the themes that are relevant to quality of life for carers and the wider family. Eligible studies for the review were primary studies of any study design that reported outcomes or themes relevant to quality of life for either people with DMD, their families, or both. A review of studies identified from searching medical bibliographic sources between 2010 and 2016 found 45 relevant published studies. A thematic framework is proposed to categorise the themes identified into: i. physical; ii. psychological; iii. Social; iv. well-being domains. A final “other” domain was included to encompass themes identified from the literature that are not covered by commonly used quality of life instruments. The rich variety of themes identified from the review highlights that DMD has a complex quality of life profile which is not currently captured by standard quality of life tools that are commonly employed in the healthcare setting. The findings also highlight that the resulting impact on the quality of life of carers and wider family of people with DMD requires consideration.

**Electronic supplementary material:**

The online version of this article (10.1186/s12955-018-1062-0) contains supplementary material, which is available to authorized users.

## Introduction

Duchenne muscular dystrophy (DMD) is a rare, inherited neuromuscular disorder affecting boys and men with an estimated incidence of 1:3800 to 1:6200 among boys born alive [1]. The disease is characterised by a progressive degeneration of muscle fibres, which results in muscle weakness. Disruption to daily life can begin as early as aged 3 years, with impact upon daily activities [2]. DMD is incurable and life expectancy is limited, with the average being reported as between approximately 23 and 28 years of age [3]. Gait loss and functional dependence typically occur in the second decade of life [3]. It is recognised that DMD impacts not only the individual, the loss of functional independence is reported to affect quality of life for both children and their families [4–8].

Literature reporting quality of life (QoL) of children with DMD is conflicting. Some studies report reduced QoL [2, 7], whilst others find no difference between QoL of children with DMD and healthy children [9–11]. However, it is possible that the findings of such studies were influenced by the way in which QoL was measured within the studies themselves. QoL and health-related QoL (HRQoL) are used interchangeably in the literature, despite being different constructs. QoL is a broad multidimensional concept which is defined by the World Health Organisation as encompassing the person’s physical health, psychological state, personal beliefs, social relationships and their relationship to salient features of their environment. Within the context of clinical studies, instruments that actually assess functional ability and/or life satisfaction scales, are often used as a measure of QoL. These instruments frequently study HRQoL which represents the patient’s general perception of the effect of illness and treatment on physical, psychological, and social aspects of life. Whilst HRQoL is a multi-domain concept, it may not adequately reflect the full scope of the impact the condition has upon the individual and their overall QoL. A recent study by Wei et al. (2017) [12] found from a study of parents and children with DMD that QoL and HRQoL are related but distinct concepts and that factors outside of ‘health’ contribute to overall QoL in the paediatric population with DMD. Currently the full range of factors that affect QoL in DMD are unknown.

The perspective from which QoL is measured may also differ. Parent proxy-reports can supplement child self-reports of QoL when a child is too young or unable to complete the assessment. However, there are recognised differences between self and proxy reporting across a range of health conditions relevant to DMD, with parents of children with disabilities tending to report lower HRQoL for their children than the children do for themselves [13–16]. The perspectives of carers for their child and also for their own QoL are therefore also valuable to understanding a more complete picture of the themes underlying QoL.

Currently no condition-specific QoL preference-based measure or conceptual model of QoL in DMD exists for this population. The purpose of this study is to identify what QoL themes are relevant to patients and families of patients with DMD reporting the use of various QoL or HRQoL instruments in this condition in recent published academic literature. “Themes” may be defined as factors that are noted to impact on QoL or noted as being relevant to QoL.

## Materials and methods

### Search strategy

Searches of MEDLINE (including Medline in Process via OVID), EMBASE (via OVID), and CINAHL (via EBSCO) medical electronic databases were conducted to identify evidence relevant to QoL in people with DMD as well as the effects on family and carers. In order to review the relevant evidence within allocated time constraints, abbreviations to comprehensive review techniques were made including limiting searches to studies published in the English language and those published between 2010 and 2016. In each database, search terms for DMD were combined with four different search statements to identify a variety of research designs. Free text and thesaurus terms were used in combination to maximise sensitivity in the searches using filters. A sample search strategy is provided in Additional file 1.

Titles and abstracts of references retrieved from the searches were assessed for inclusion against a priori established eligibility criteria by one reviewer. Reference lists of included studies were manually searched for further relevant studies that were not captured by the searches. Table 1 outlines the inclusion and exclusion criteria used to screen studies that would be eligible to the review. Studies of interest included primary research studies of people with DMD investigating issues related to the patient, the carer or the family’s QoL. This included research designs such as surveys, questionnaires, clinical trials, qualitative research or other designs of QoL research.

**Table 1:** Eligibility criteria for studies included in the review

Inclusion criteria	Exclusion criteria
• Patients: Studies of adults and children of any age with a confirmed diagnosis of DMD, and/or of families and carers of people with DMD • Intervention/Exposure: Measures of quality of life • Comparator: Not applicable • Outcomes: Quality of life • Studies: Studies using QoL instruments or qualitative studies are the primary design of interest. Cross-sectional or cohort studies reporting QoL are eligible. Trials of patients with DMD which report measuring relevant QoL data in study abstracts will be considered for inclusion. • Studies published after 2010	• Studies of patients with other forms of MD • Discussion articles, reviews or editorials without study data • Studies published in non-English language • Studies published prior to 2010 • Observational studies of aetiology or onset • Studies which are not discussed in relation to QoL • Studies regarding screening for DMD without reference to QoL • Animal studies • Phase 1 clinical studies or lab studies • Letters and commentaries • Citation titles without abstracts • Case reports

### Data synthesis

Studies selected for inclusion were read in full, annotated and coded to extract themes relevant to QoL from the results and discussions sections. One reviewer performed data extraction for QoL themes without blinding to study author. Themes were then pooled across studies into a master database and grouped firstly into domains: i) Physical; ii) Psychological; iii) Social; iv) Well-being as these are commonly encountered domains in general QoL research. Sub-domains within each domain were then created to capture the variety of data from the included studies. Themes that did not fall under any of the four initial domains were grouped under an “Other” domain. This framework of themes was reviewed by one further review author to verify agreement of coding. As the review is not an aggregation of data to provide a pooled treatment effect, or to rank findings based on frequency, no quality assessment or risk of bias assessment was deemed necessary.

## Results

**Fig. 1:**
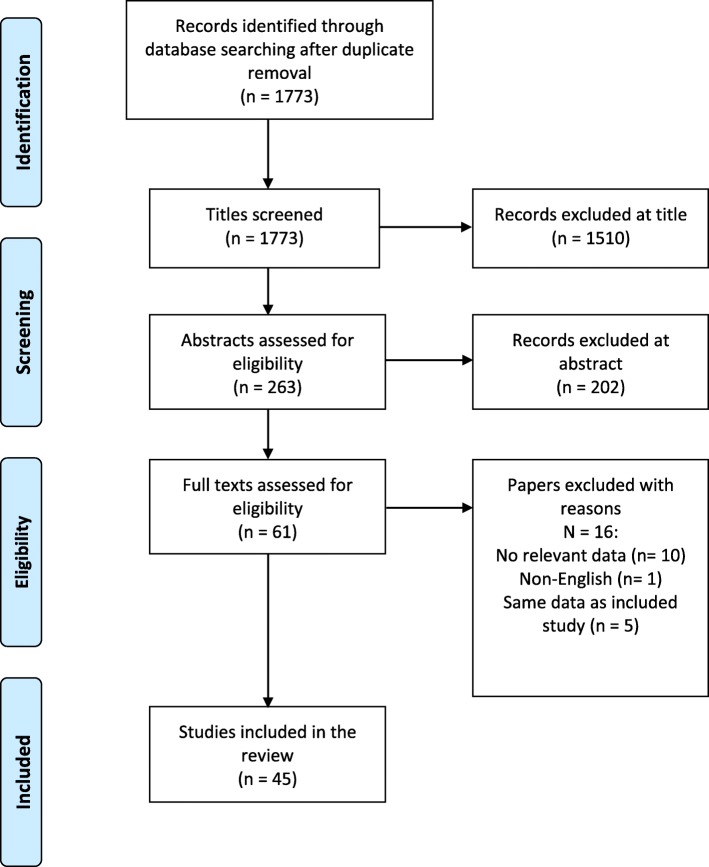
Flow Diagram of Studies Identified and Included in the Review

The literature searches yielded 1773 records after duplicates were removed. Figure 1 documents the selection of studies at title and abstract stage. Titles and abstracts were reviewed against eligibility criteria (Table 1) to identify potentially relevant full text articles. A total of 45 studies were identified for inclusion into the review. No further studies were identified from searching reference lists of included studies indicating that the restrictions to English language papers and those published after 2010 are unlikely to have limited the validity of the bibliographic searches.

### Included studies

Table 2 provides the details of the 45 included studies, published from 2010 to 2016. Three studies were conducted in the UK whilst another four were conducted in the UK in combination with other European countries. Nine were conducted in the United States, 16 in European countries and 13 from other countries around the world. The number of participants (N) in the studies ranges from 10 to 770 with a median of 60. Cross-sectional studies of DMD patients or carers were the most common study design (*n* = 27), followed by case-control (*n* = 8), cohort/before and after studies (*n* = 6), tool development only (*n* = 2). There was one mixed-methods study (audit, interviews and survey) and one qualitative study. The majority of studies focused on children and adolescents with DMD (*n* = 30) whilst nine studies were of parents/ carers of people with DMD. Six studies were interested in both patient and parent/carer perspectives.

**Table 2:** Included Studies

First author	Study type	Data source	N	Country	Method of quality of life measurement
Baiardini et al.*,* 2011 [7]	Cross sectional survey	DMD boys & families	27	Italy	Parent-proxy HRQoL
Bendixen et al.*,* 2012 [2]	Case-control survey	DMD boys & controls	50	US	Self-reported QoL
Bendixen et al.*,* 2014 [60]	Cross sectional survey	DMD boys by age	60	US	Self-reported participation in daily activities
Bloetzer et al.*,* 2012 [61]	Cross sectional survey	DMD boys	63	Switzerland	Parent-proxy sleep disturbances
Bray et al.*,* 2011 [62]	Cohort survey, 6 month follow up	DMD boys & controls	34	Australia	Parent-proxy HRQoL
Bray et al.*,* 2010 [8]	Cross sectional survey	DMD boys & parents	35	Australia	Self-reported & parent-proxy HRQoL
Cavazza et al.*,* 2016 [56]	Cross sectional survey	Patients or caregiver	422	UK & 7 European countries	Self-reported or parent-proxy HRQoL
Dany et al.*,* 2015 [35]	Tool development	NMD patients	159	France	Self-reported HRQoL
Davison et al.*,* 2011 [28]	Qualitative study	DMD & other NMD adolescents	13	UK	Self-reported social well being
Elsenbruch et al.*,* 2013 [20]	Cross sectional survey	DMD children, adolescents & adults	50	Germany	Self-reported HRQoL
Garralda et al.*,* 2013 [30]	Cohort study	DMD boys	19	UK	Emotional impact of gene-modifying trial
Geers et al.*,* 2011 [55]	Case-control survey	DMD children, adults & matched controls	36	Germany	Self-reported HRQoL
Heutinck et al.*,* 2015 [27]	Case-control survey	DMD boys & healthy controls	86	Holland	Physical activity [respondent not reported]
Houwen et al.*,* 2014 [40]	Cross sectional survey	DMD boys & parents	40	Holland	Self-reported & parent-proxy HRQoL
Hunt et al.*,* 2016 [17]	Cross sectional interviews & survey	DMD boys & adult & parent pairs	12	UK	Self-reported & parent-proxy QoL
Klingels et al.*,* 2016 [31]	Tool development	Systematic review & expert opinion	194	Belgium	Activities of daily living
Landfelt et al.*,* 2016 [37]	Cross sectional multi-national survey	DMD patients & parents & general population reference values from Canada	770	UK, Europe & USA	Self-reported & parent-proxy HRQoL
Landfelt et al.*,* 2016 [32]	Cross sectional survey	Caregivers & general population reference values from Canada	770	UK, Europe & USA	Caregiver HRQoL & burden
Lim et al.*,* 2014 [63]	Cross sectional survey	DMD boys & parents	63	US	Self-reported & parent-proxy HRQoL
Lue et al.*,* 2016 [54]	Cross sectional survey	DMD adolescents & adults	46	Taiwan	HRQoL & global QoL [respondent not reported]
Ly et al.*,* 2013 [21]	Cross sectional survey	DMD adults	10	US	Self-reported or proxy social & medical support
Madsen et al.*,* 2014 [22]	Cross sectional survey	DMD adults	79	Denmark	Self-reported QoL
Magliano et al.*,* 2015 [44]	Cross sectional survey	Relatives of MD patients	502	Italy	Caregiver burden
Magliano et al.*,* 2014 [19]	Case-control survey	DMD parent vs Becker parent	246	Italy	Self-reported caregiver burden
Martinsen et al.*,* 2012 [23]	Cross sectional interview	DMD adults	16	Denmark	Self-reported dependence on care
Mason et al.*,* 2016 [64]	Cross sectional survey	DMD adults	29	Australia	Self-reported QoL
McSweeney et al.*,* 2011 [65]	Cross sectional survey	DMD children	39	Ireland	Self- reported & parent-proxy HRQoL
Messina et al.*,* 2016 [34]	Cohort survey, 12-month follow up	Ambulatory DMD	98	Italy	Self-reported & parent-proxy HRQOL
Nozoe et al.*,* 2014 [66]	Cross sectional survey	Mothers of DMD children & control mothers	20	Brazil	Parent sexual function
Ozyurt et al.*,* 2015 [46]	Case-control study	DMD, parents & controls	17	Turkey	Self-reported, parent-proxy QoL & parent anxiety
Pangalila et al.*,* 2015 [42]	Cross sectional survey	DMD adults	80	Holland	Patient HRQoL [respondent not reported]
Pangalila et al.*,* 2012 [33]	Cross sectional study	Parents of severely disabled DMD adults	80	Holland	Subjective caregiver burden
Peay et al.*,* 2016 [67]	Cohort survey, 2 yr. follow up	Mothers of DMD children	205	US	Caregiver burden
Peay et al.*,* 2013 [26]	Cross sectional survey	Parents of DMD children	119	US	Caregiver burden
Reha et al.*,* 2014 [25]	Cohort survey	DMD patients	47	US	Self-reported or parent-proxy QoL
Riss et al.*,* 2012 [68]	Cross sectional survey	DMD boys	25	US	Chart review & parent-proxy QoL
Simon et al.*,* 2011 [11]	Cohort survey, 1 year	DMD patients	95	Brazil	QoL during steroid therapy [respondent not reported]
Soares et al.*,* 2015 [45]	Cross sectional survey	DMD children & caregivers	35	Brazil	Patient QoL Caregiver QoL
Steffensen et al.*,* 2015 [24]	Cross sectional survey	DMD adults	183	UK & 6 European countries	Self-reported HRQoL
Thomas et al.*,* 2014 [29]	Cross sectional interview	Caregivers	60	India	Caregiver burden
Uzark et al.*,* 2012 [43]	Case-control survey	DMD boys, parents & matched controls	117	US	Self-reported & parent-proxy HRQoL
Wei et al.*,* 2016 [39]	Cross sectional survey	Families of DMD boys	176	Canada	Parent-proxy HRQoL
Wei et al.*,* 2014 [41]	Case-control survey	DMD children	176	Canada	Self- report & parent-proxy HRQoL
Wong et al.*,* 2015 [18]	Audit, interviews & survey	Clinical records & parent survey	49	Australia	Parents’ experiences from first noticing symptoms to receiving a diagnosis
Zamani et al.*,* 2016 [69]	Case-control survey	DMD boys, parents & healthy controls	85	Iran	Self-reported & parent-proxy QoL

Most studies reported measuring QoL or health-related QoL as the primary outcome of interest. However, some studies were not strictly investigating QoL but did study and report issues that could be of potential relevance to QoL. A variety of named questionnaires and instruments were employed across the studies (*n* = 36) with 18 studies employing multiple instruments. Five studies employed both generic instruments and a specific instrument for paediatric neuromuscular disorders [17–21]. A condition-specific instrument for QoL in DMD does not currently exist. Table 3 documents the instruments reported across the 45 included studies.

**Table 3:** Survey scales, Questionnaires or Instruments Used across Studies

Instrument Used	No. of studies
Pediatric Quality of Life Inventory (PedsQL) neuromuscular module version 3.0 online [2, 8, 17, 19, 39, 41, 44, 60, 61]	9
SF-36 Health Survey [11, 28, 43, 54, 55, 62]	6
World Health Organization Quality of Life (WHOQoL) Scale [28, 43, 46, 54, 62]	5
KIDSCREEN questionnaire (child and adolescent version and parent version) [42, 63]	2
EuroQol (EQ-5D 3 L) [20] (EQ-5D 5 L) [56]	2
Zarit Caregiver Burden Interview (ZBI) [20, 46]	2
Pittsburgh Sleep Quality questionnaire (PSQI) [17, 64]	2
Children’s Assessment of Participation and Enjoyment (CAPE) [2, 65]	2
Child Health Questionnaire parent form (CHQ-PF50) [7, 66]	2
Health Utilities Index Questionnaire (HUI) [39]	1
SF-12 Health Survey [20]	1
Fatigue Severity Scale [43]	1
Hospital Anxiety and Depression Scale (HADS) [43]	1
Barthel Index (BI) [45]	1
Family Problems Questionnaire (FPQ) [45]	1
Muscular Dystrophy Care Schedule (MD-CS) [45]	1
Female Sexual Function Index (FSFI) [64]	1
Sleep Disturbance Scale for Children (SDSC) [68]	1
Caregiver Strain Index (CSI) [36]	1
Self-Rated Burden (SRB) [36]	1
Care-related Quality of Life instrument (CarerQol) [36]	1
Family Strain Questionnaire [7]	1
Family Burden Assessment Scale [33]	1
COPE Inventory (60-item) [33]	1
Caregiver Well-Being Scale [33]	1
Quality of Life in Neuromuscular Disease (QoL-NMD) [37]	1
DISABKIDS Questionnaire for 10–16 years [55]	1
Depressionsiventar fur Kinder und Jugendliche (DIKJ) [55]	1
Beck Depression Inventory (BDI) [55]	1
CARE-NMD questionnaire [62]	1
State-Trait Anxiety Inventory (STAI) [17]	1
validated pediatric QOL survey (Still in progress) [67]	1
Modified Brooks Scale (MBS) [67]	1
Life Satisfaction Index for Adolescents (LSI-A) [11]	1
Quality of Life Evaluation Scale (AUQUEI) [11]	1
Activity Limitations (ACTIVLIM) [65]	1

A total of 36 validated instruments were used across the 45 studies. The most commonly employed instruments were the Pediatric Quality of Life Inventory (PedsQL) (*n* = 9), the Short Form-36 (SF-36) (*n* = 6), and the World Health Organization Quality of Life (WHOQoL) (*n* = 5). A small proportion of studies did not provide specific details for the instruments used (*n* = 7) [18, 22–27] or they used non-validated questionnaires or interview schedules (*n* = 8) [28–35] or visual analogue scales (*n* = 2) [20, 36].

### QoL domains identified from the included studies

Three of the included studies focussed mainly on the development of instruments measuring QoL in the patient population [21, 35, 37]. The remaining 42 studies covered a large variety of topics relevant to people with DMD and their carers’. Themes from all 45 studies were extracted and coded into the following five domains: i) Physical; ii) Psychological; iii) Social; iv) Well-being and; v) Other. The first four domains were derived from an initial a priori framework to encompass the commonly anticipated domains in general QoL research. Based on the number, variety and recurrence of themes identified across the included literature, the domains were further divided into relevant sub-domains as presented in Table 4. An “other” domain was created to capture themes recurring and potentially relevant that may be regarded independently to the four initial domains. Themes were assimilated across studies into one framework to encompass and summarise the diversity of research.

**Table 4:** Quality of Life Domains and Sub-Domains Identified Across the Included Studies

Physical Domain 1. General physical QoL 2. Health behaviour 3. Sleep 4. Pain 5. Activities of daily living	
Social Domain 1. Social QoL 2. Participation 3. Friends 4. Relationships 5. School / work	
Psychological Domain 1. General psychosocial QoL 2. Happiness 3. Depression 4. Anxiety 5. Coping 6. Communication	
Well-Being Domain 1. General well-being 2. Independence / self-care 3. Dignity 4. Energy / fatigue	
Other Domain 1. Accessibility / wheelchair use 2. Healthcare service provision (impact related to access to formal care, medical aids etc) 3. Treatment related / therapy effects 4. Family resources (financial) 5. Carer burden 6. Impact on wider family (family functioning, siblings etc)	

The domains and sub-domains were derived from either studies measuring self-reported QoL of people with DMD, parent proxy estimations, parent/carer/family specific or general public estimations. Themes are presented by domain and sub-domain for people with DMD in Tables 5 and carers of people with DMD in Table 6.

**Table 5:** Themes and Trends Identified Under the Physical, Psychological, Social, Well-being and Other Domains for DMD Patients

Physical Functioning Themes	
General physical QoL	Impaired QoL according to decline in ambulatory status [54]
	Impaired QoL reported (according to both self-report [44], and parent-proxy report [44, 60]
	Self-report higher than parent [8]
	Declines with age [19, 22, 61]
	Impaired compared to controls/normative data [24, 42, 55, 62]
	Varies according to geographical status [28]
	Impairment correlates with anxiety [43]
	Impairment correlates with wheelchair or ventilator use [7, 19]
	Physical functioning QoL improvement with treatment [29]
	Fractures occur more frequently in more ambulant stages [63]
	Physical activity lower than for age-matched controls [2, 31, 60]
Health Behaviour	Physical activity less strenuous than age-matched controls [31]
	Physical activity decline with age by self-report [64]
	More on-screen/sedentary behaviour with age [31]
	Physical activity improvement with treatment by self-report or parent proxy [29]
Sleep	Sleep quality lower than controls by self-report or parent proxy [17]
	Problems initiating and maintaining sleep (DIMS), sleep-related breathing disorders and sleep hyperhidrosis by parent-proxy [65]
Pain	Pain correlates with reduced QoL [22]
	Occurrence of pain not reflected in associations of general QoL [43]
	Pain complaints largely kept within the family [22]
	Physical abilities restrict pain-coping strategies [22]
Activities of Daily Living	Problems with day-to-day practicalities compounds problems in other domains [32]
	Daily activities such as transportation to school are passive for majority of patients whereas controls use active transport [31]
Social Themes	
General Social QoL	Lower QoL for social domain than unaffected boys [2, 19, 24, 32, 55, 61, 62]
Participation	Adolescents expressed longing for missed activities [44, 54]
	Children perceive their ability to keep up with their peers as less difficult than their parents do [67]
	Inability to participate in activities with peers further aggravates social problems [24, 44, 54]
	Lack of correlation between decrease in participation and general QoL [2]
	Low level of participation leads to life devoid of meaningful activities [43]
	Social activities and participation not correlated with “social relationships” [37]
	Decline of social participation with increasing age [2, 64]
	Significantly more time spent on screen time activities that controls [31]
	Decrease in participation correlates with time to walk up stairs and decrease in physical activities [64]
Friends	Adolescents expressed longing for missed friends [32, 54]
	Children and parents rate their children as having lower QoL regarding “friends” than controls [44, 60]
	Parents perceive lower HRQoL for social acceptance than their sons self-report [42]
	Accessibility to homes becomes a physical barrier to visiting friends [27]
	Carers have crucial role in enabling patients to see family and friends [27]
Relationships	Score low in the domain of social relationships compared to reference population [32, 43]
	Few patients had expectations of successful future relationships [32]
School/Work	Most common school problem was missing school to go to doctor or hospital [44]
	Parent report more school days missed because of not feeling well than their children [67]
	No difference to controls for “school-related” QoL [2]
	Parents report practical difficulties with sending their child to school [33]
	Hopes for future employment and education attenuated by lack of independence and difficulties accessing work experience [32]
Psychological Themes	
Psychosocial QoL	Psychosocial QoL lower than general paediatric public([7], #1851, [19, 44])
	Older patients did not tend to perceive lower psychosocial QoL despite increased physical limitations [44, 55]
	Patients receiving corticosteroids report no difference in psychosocial QoL compared with patients not receiving steroids [44]
	Mental health QoL varies according to geographical status (better in North Western than Eastern Europe) [28]
	Family income associates with better Generic Core Psychosocial score [19]
Happiness	Most patients rated as happy and in good health by caregivers compared to public preference which estimates substantial impairment [39]
	Parents rated their children lower for “general mood” and feelings than control parents [42, 60]
	Parents rated their children lower for “moods and emotions” than their children’s self-report [42]
Depression	Depressive symptoms were in the subclinical range and did not correlate with physical disability [24]
Anxiety	Correlated with overall QoL and with physical health and psychological functioning [43]
	Patients worried about their future and about their family [44]
Coping	Emotional impact of trial participation mediated by baseline psychosocial stressors [34]
	Communication with family and friends is an important coping mechanism [44]
	Coping mechanisms posited as reason for maintenance of psychosocial QoL in older boys [55]
Communication	Communication difficulties mean patients not always able to provide self-assessments [39, 66]
	Parents underestimate their child’s HRQoL compared to self-report [67]
	Motor impairments mean dependence on help to complete assessments may introduce reporting bias [19, 24, 44]
	Dependence on help to complete assessments may inhibit respondents from admitting extent of feelings [24, 44]
	Patients often found it difficult to talk to non-medical people [44]
	Patients rely on familiar people to be able to communicate effectively [22, 27, 44]
	Well-Being Themes
General Well-Being	Perceived HRQoL underestimated by parents compared to self-perception by self-report [42]
Independence/ Self-Care	Parents help required to complete self-assessment [39]
	Patients become increasingly dependent on parent/carer with age [26, 27, 37]
	Patients dependent on parents and carers to act on their behalf to relieve pain [22]
	When patients are less able to take care of themselves independently, they perceive their physical abilities lower [42]
	Parents help required to complete self-assessment [39]
	Patients become increasingly dependent on parent/carer with age [26, 27, 37]
Dignity	Many patients consider their QoL as good and feel respected by society [26]
	Creative engagement and hobbies important to feelings of identity and autonomy [27]
	Attitudes to accessibility are of great importance to patient’s integration in society [19]
	Importance of patient’s needs and wishes as an individual to be respected [27]
Energy/Fatigue	Less fatigue correlates with better QoL [19, 41]
	Low QoL for fatigue reported by patients [42, 43]
	Lower QoL for fatigue reported by patients than by patients [67]
Other Themes	
Accessibility/ Wheelchair Use	Use of electric wheelchairs can promote participation in activities [54]
	Affordable access to medical devices is central to maintenance of QoL as physical functioning deteriorates [39]
	Access to public transport and access to the professional world were barriers to participation [32, 37]
	Intermittent wheelchair use associated with greater fatigue than children not using a wheelchair [19]
	Wheelchair use correlates with poorer QoL [7, 19]
	Lack of wheelchair access to premises can be a barrier to participation and social activities [27]
Healthcare Service Provision	Earlier diagnosis benefits include ability to access specific treatments sooner, preparing for financial and practical issues in the future and informing reproductive planning [23]
	Large proportion of carers report adequate professional support [45]
	Medical (e.g. splints, shower chairs) and leisure equipment (PlayStation) can exacerbate or influence pain [22]
	Choices of equipment, seating, beds and routes of medication should be available to carers [22]
	Patients felt nurses were sometimes not adequately trained in ventilator care [25]
	Lack of knowledge from primary healthcare providers on the specific complications of DMD [25]
	All adult patients (> 20 yrs.) required help around the clock [26]
	Main driver of non-healthcare costs to the healthcare system is informal care [56]
Treatment Related/ Therapy Effects	Some medications were difficult for patients to swallow whilst others had side effects [22]
	Providing pre-emptive analgesics and adjunctive medication for common side effects alongside should be considered [22]
	Potential emotional impact of trial participation on patient and family [34]
	Higher incidence of fracture for patients on steroids [63]

**Table 6:** Themes and Trends Identified Under the Physical, Psychological, Social, Well-being and Other Domains for Carers

Carer: Physical Functioning Themes	
General Physical QoL	Correlates with burden [8, 18, 45, 60]
Sleep	Poor quality of sleep correlates with hormonal changes related to stress [61]
	Poor quality of sleep correlates with sexual dysfunction [61]
Carer: Psychological Themes	
Happiness	Indicate similar levels of happiness to the general population [36]
Depression	PARENT: Correlated with annual household cost burden and hours of leisure time devoted to informal care per week [20] High number of relatives report feelings of depression [45]
	Patient’s loss of ambulation reported as most emotionally difficult time for parents [62]
	Parents report feelings of loss about child’s condition [18, 45]
Anxiety	Correlated with annual household cost burden and hours of leisure time devoted to informal care per week [20]
	No difference between anxiety levels of patient mother versus controls [17]
	High number of relatives report feeling worry about future of other family members [45]
	Higher anxiety correlated with less active coping style [36]
	Parents prioritised the worries about affected children and their care before worries regarding themselves [30]
Coping	Psychological adaptation to DMD predicted by resilience [63]
	Parents felt unable to bear the situation longer compared to parents of Becker MD [18]
	Active coping style correlates with lower caregiver burden [36]
	Emotional coping was most disrupted around the time of loss of ambulation [62]
	Coping strategies such as positive reinterpretation and religion correlate with understanding of the illness [33]
	Social support an important coping strategy [33]
Communication	Difficulties communicating about the condition with affected sons [33]
Carer: Social Themes	
Participation	Constraints in leisure activities and neglect of hobbies frequently mentioned concern from parents [18]
	Perception of stigma in a public setting [18]
Friends	Psychological burden higher in those with lower social contacts and support from friends/relatives in emergencies [18]
Relationships	Psychological burden higher for parents without a cohabiting partner [45]
	Parents report interpersonal issues or problems in family functioning [33]
Carer: Well-Being Themes	
General Well-Being	Caregiving described not only as a burden but as an important, rewarding activity [18, 33, 36]
	Caregiver well-being rated as moderate [33]
Carer: Other Themes	
Accessibility/ WheelcHair use	Patient’s transition to wheelchair reported as most emotionally difficult time [62]
Treatment Related/	Parental concern about getting right treatment for child and missing out on new treatments [30]
Family resources	Family income restricts abilities to care for son [20, 30, 33, 45]
	Higher family income correlates with better HRQoL [19, 20, 36]
Carer Burden	Higher perceived caregiver burden correlated with worse functional status in the child [18, 44, 45, 63]
	Higher psychological burden in those who did not live with a partner [45, 64]
	Practical burden correlates with daily time in taking care of the patient [33, 45]
	Higher burden in relatives of DMD patients compared with LGMD and BMD [45]
	High number of relatives report feeling guilt for having transmitted the illness to their children [45]
	Burden correlates with duration of illness [18, 62, 65]
	Burden higher among those with fewer social contacts and lower social support in emergencies [18, 33]
	Parent concerns about quality of care their sons received and problems with physical distance if/ when their sons leaves home [36]
	Relates to time caring for children not functional dependence [46]
	Carer age has no correlations with other factors relevant to burden [62]
Impact on Wider Family	DMD parents are significantly different to Becker MD parents for feelings of stigma and neglect of hobbies [18]
	Parents believed MD has a negative influence on the psychological well-being, and social life of unaffected children [18]
	Difficulties among healthy siblings reported as higher by parents who were older, had higher burden and lower social contacts [18]
	Support from own social contacts relied upon in event of carer illness [45]
	Substantial differences between DMD and BMD caregivers ability for night waking; neglect of hobbies; work/household difficulties; taking holidays and financial difficulties [18]
	Substantial number of parents believed patient’s condition negatively influenced psychological well-being of unaffected children [18]
	Fathers reported lower levels of satisfaction with the family relationship than mothers [7]
	Some parents reported interpersonal issues of problems in family functioning [33]
	Substance use reported to be higher among male caregivers [33]
Carer: Physical Functioning Themes	
General Physical QoL	Correlates with burden [19, 44, 62, 70]
Sleep	Poor quality of sleep correlates with hormonal changes related to stress [66]
	Poor quality of sleep correlates with sexual dysfunction [66]
Carer: Psychological Themes	
Happiness	Indicate similar levels of happiness to the general population [33]
Depression	PARENT: Correlated with annual household cost burden and hours of leisure time devoted to informal care per week [32] High number of relatives report feelings of depression [44]
	Patient’s loss of ambulation reported as most emotionally difficult time for parents [62]
	Parents report feelings of loss about child’s condition [19, 44]
Anxiety	Correlated with annual household cost burden and hours of leisure time devoted to informal care per week [32]
	No difference between anxiety levels of patient mother versus controls [46]
	High number of relatives report feeling worry about future of other family members [44]
	Higher anxiety correlated with less active coping style [33]
	Parents prioritised the worries about affected children and their care before worries regarding themselves [26]
Coping	Psychological adaptation to DMD predicted by resilience [67]
	Parents felt unable to bear the situation longer compared to parents of Becker MD [19]
	Active coping style correlates with lower caregiver burden [33]
	Emotional coping was most disrupted around the time of loss of ambulation [62]
	Coping strategies such as positive reinterpretation and religion correlate with understanding of the illness [29]
	Social support an important coping strategy [29]
Communication	Difficulties communicating about the condition with affected sons [29]
Carer: Social Themes	
Participation	Constraints in leisure activities and neglect of hobbies frequently mentioned concern from parents [19]
	Perception of stigma in a public setting [19]
Friends	Psychological burden higher in those with lower social contacts and support from friends/relatives in emergencies [19]
Relationships	Psychological burden higher for parents without a cohabiting partner [44]
	Parents report interpersonal issues or problems in family functioning [29]
Carer: Well-Being Themes	
General Well-Being	Caregiving described not only as a burden but as an important, rewarding activity [19, 29, 33]
	Caregiver well-being rated as moderate [29]
Carer: Other Themes	
Accessibility/ Wheelchair Use	Patient’s transition to wheelchair reported as most emotionally difficult time [62]
Treatment Related/	Parental concern about getting right treatment for child and missing out on new treatments [26]
Family Resources	Family income restricts abilities to care for son [26, 29, 32, 44]
	Higher family income correlates with better HRQoL [32, 33, 41]
Carer Burden	Higher perceived caregiver burden correlated with worse functional status in the child [19, 43, 44, 67]
	Higher psychological burden in those who did not live with a partner [44, 61]
	Practical burden correlates with daily time in taking care of the patient [29, 44]
	Higher burden in relatives of DMD patients compared with LGMD and BMD [44]
	High number of relatives report feeling guilt for having transmitted the illness to their children [44]
	Burden correlates with duration of illness [19, 62, 65]
	Burden higher among those with fewer social contacts and lower social support in emergencies [19, 29]
	Parent concerns about quality of care their sons received and problems with physical distance if/ when their sons leaves home [33]
	Relates to time caring for children not functional dependence [45]
	Carer age has no correlations with other factors relevant to burden [62]
Impact on Wider Family	DMD parents are significantly different to Becker MD parents for feelings of stigma and neglect of hobbies [19]
	Parents believed MD has a negative influence on the psychological well-being, and social life of unaffected children [19]
	Difficulties among healthy siblings reported as higher by parents who were older, had higher burden and lower social contacts [19]
	Support from own social contacts relied upon in event of carer illness [44]
	Substantial differences between DMD and BMD caregivers ability for night waking; neglect of hobbies; work/household difficulties; taking holidays and financial difficulties [19]
	Substantial number of parents believed patient’s condition negatively influenced psychological well-being of unaffected children [19]
	Fathers reported lower levels of satisfaction with the family relationship than mothers [7]
	Some parents reported interpersonal issues of problems in family functioning [29]
	Substance use reported to be higher among male caregivers [29]

### Important and recurring trends in the data

Particular trends in the data were noted during the course of the review. The tendency for the general public to estimate the HRQoL of people with DMD lower than the patients themselves, or the so-called *disability paradox* [38], was observed indicating the potential for preference-based measures that necessarily incorporate public views to underestimate QoL in DMD. Two studies documented substantial differences between public preference estimates for QoL in DMD compared to patients [20, 39]. These variations are in line with previous literature [38, 40] on the topic of incorporating different perspectives in health state valuation and highlight the need to fully consider the reasons behind these differences and the potential consequences of low agreement.

Agreement between patients and their carers tended to be consistent across domains. However, as noted by other research [13–16] a number of studies found that patients with DMD rate their QoL more favourably by self-report compared to their caregivers’ proxy report [19, 39, 41, 42]. A variety of reasons for this are posited across these studies. Parents own fears and worries about their child’s disease may influence their assessment of their child’s HRQoL [19]. Children may have adapted to their illness better than their parents have and parents may not always have the most accurate assessment of their child’s internal state [19]. Also, parents may reflect on their own frame of reference by interpreting the limitations of their sons or they may be more sensitive to negative comments of the environment [42]. Reasons postulated for low agreement between self- and proxy report appear most relevant to differential processes of adaptation between children and their parents.

Alternatively, if the notion that parents underestimate their child’s QoL is rejected, it is conceivable that children may have overestimated their QoL under certain circumstances. For example, one study suggested that when tests were completed with help of the paediatricians who are conducting the assessment, an element of reporting bias may be introduced [24]. Patients were noted as less likely to mention pain as one of their complaints [43]. The issue of pain, and the communication of pain, is a potentially relevant factor to the issue of differences between self and proxy reports of QoL. One study [22] reports that patients were limited in their ability to manage their own pain and may only confide in pain to parents even though these discussions might need prompting. In this respect children are dependent on their parents or carers correctly picking up on their behavioural and emotional indicators of pain. The reluctance of the young men to talk about their pain may be linked to this reliance on other people to take action on their behalf [22]. Therefore, those interested in measuring HRQoL should be aware of potential response biases that may mean that relevant factors to patients’ well-being are overlooked. The consequences of parental underestimation of their sons’ QoL could have a negative effect on decision making when boys are too ill to speak for themselves [8]. One study found that boys reported more worry than appreciated by parents which in turn may interfere with communication and the parent’s understanding of their child’s psychosocial needs [44]. Here the importance of communication is evident as well as the accuracy of parent-proxy estimations in the absence of the child being able to complete a self-report.

### Interventions called for in the literature

There were a number of suggestions for interventions required for people with DMD and their families discussed in the included studies. Whilst not directly relevant to QoL they may provide further information about which QoL domains were regarded as important to patients and their families. These included, but are not limited to: initiatives to relieve family burden and improve carer well-being [18, 20, 33, 45, 46]; social skills training and modified activities inclusive of children with limited mobility [41]; education and care focused on optimising participation [43]; strategies to avoid diagnostic delay [23]; routine screening questionnaires and vigilance for signalling symptoms of pain, depression and anxiety in patients [43]; and improvements to welfare policies to simplify bureaucratic procedures [45].

### Further research called for in the literature

Suggestions for future primary studies included research investigating the effect on other family members [17, 18, 45]; studies with follow up, rather than cross-sectional designs [17–19, 44]; studies with more diverse samples with regards to race, educational status and single-parent families [44]; and further validation of the QoL-NMD [37].

## Discussion

This study has highlighted a large variety of quantitative research and few qualitative studies investigating QoL in DMD patients and carers. A large number of themes and trends in the data were identified by the authors of the included studies as being relevant to the QoL of people with DMD, their carers’ and their families. The physical, psychological, social and well-being domains and each of their subdomains are populated by several themes, many of which are commonly already known about DMD. Some themes were identified however, which are not commonly investigated using validated instruments, particularly those under the “other” domain. These themes highlight the complexity and uniqueness of the DMD as they relate to the importance of accessibility, communication, particularly regarding proxy versus self-report, the relationship to and impact upon the carer, and also the importance of appropriate healthcare services or family resources. These themes are unlikely to be adequately assessed for DMD patients by generic preference-based QoL instruments.

### Understanding the range of impact to QoL from DMD

This review included studies that used either quantitative and/or qualitative methods to determine the impact of DMD on QoL. However, there were very few qualitative studies identified, with most studies using an instrument to measure the impact of DMD on QoL. The included studies employed a wide range of different instruments that are relevant to QoL. This is common in the investigation of QoL, particularly for children [47]. Given complexities specific to DMD, the difficulties of using a generic HRQoL instrument in a specific disease area were acknowledged. Baiardini et al (2011) [7] suggested it would be impossible to use the same measure of HRQoL for all DMD patients due to the age range of affected patients. Another study which employed the QoL-NMD, an instrument specific to neuromuscular disease, even reported that that were some categories that were covered by a more generic scale (the INQoL) that were not covered by the more disease-specific QoL-NMD [37]. These authors recommended that a questionnaire specific to each neuromuscular disorder should be developed on the basis that whilst genetic neuromuscular disorders share a common pattern (i.e. a progressive loss of physical condition), they are also heterogeneous on several criteria. These include the age of disease onset, muscles affected, and the range of severity between the beginning and the end of the disease. Therefore, DMD can be considered as quite unique within the broader category of neuromuscular dystrophy.

### Relevance and validity of instruments used in the studies

A key benefit of using generic HRQoL instruments is the comparability of findings across clinical areas, for example, to estimate cost-effectiveness of interventions. Commonly employed generic HRQoL instruments such as the SF-36 and WHOQoL-BREF were used with different levels of success in the included studies. Landfeldt et al (2016) found that the SF-12 indicated that DMD patients had normal physical health where the EQ-5D did not [20]. Pangalila et al (2015) found that the occurrence of pain, anxiety and depression were not reflected in the QoL results using the WHO-QoL BREF and that only the ‘social relationships’ domain of QoL was impaired compared to the reference population [43]. Houwen-van Opstal et al (2014) highlights that the PedsQL, which is the most frequently used instrument in this review, was not designed to assess the full range of functioning unlike KIDSCREEN-52 [42]. The relationship between QoL and physical functioning is noted to be a complex, non-linear one with QoL for both children and/or carers potentially improving following the loss of ambulation where the risk of children falling is removed and the use of adaptive medical aids becomes routine. The same authors also highlight that there is no specific outcome measure to assess participation levels in patients. This issue was seen to be particularly lacking for people with DMD and their families. They also note that the use of generic HRQoL instruments sometimes means the inclusion of some inappropriate questions, such as “were you able to run well?” for patients with DMD.

The included literature identified varied aspects of physical, psychological and social implications of DMD. As stated, these were primarily measured using quantitative methods via existing QoL instruments. Table 3 shows that the common instruments used were PedsQL (neuromuscular module), SF-36, and WHOQOL. However, it can be argued that the SF-36 and WHOQOL instruments are not necessarily appropriate for the DMD population, as being that these instruments are generic, they do not incorporate all factors that are relevant to QoL for people with DMD. The PedsQL Neuromuscular Module (PedsQL-NMD) is a specific module of the PedsQL. Existing versions of this questionnaire include Acute version and Standard version, for Toddlers (2–4 years of age), Young Child (5–7 years of age), Child (8–12 years of age), Adolescent (13–18 years of age). The neuromuscular module was developed for neuromuscular disorders such as spinal muscular atrophy (SMA), DMD and other chronic conditions. Whilst this instrument may contain items that are relevant to those with DMD, it should be noted that the construct of the PedsQL-NMD was developed with participants aged from 18 to 80 years.

### Important aspects of QoL to DMD

The literature suggests that many aspects of QoL are important to those with DMD, but the majority of these have been determined in a top-down fashion via existing instruments. It is possible that the incorporation of additional or alternative QoL themes into QoL assessment in DMD using a bottom-up approach with the choice of a wider choice of themes could benefit the study of QoL in this specific condition. Qualitative studies that build upon the themes identified in this review, undertaken with children and adolescents with DMD may identify new themes, or highlight QoL themes that are not relevant.

### The importance of age

Age is an important factor to consider when measuring the impact of any condition upon QoL. The degree to which a child can self-complete a questionnaire is dependent upon their ability. Young children may require assistance with reading the questionnaire, and the administrative procedures. The task burden (which incorporates both the complexity of the questionnaire, and the number of items within it) will influence the quality of the data an instrument is able to provide. Instruments that contain a large number of items may mean that younger children are unable to reliably complete them. Matza et al. (2004) [48] identified a number of considerations to be made when designing a paediatric QoL instrument. The first is the age at which children can report their own health. Children appear able to reliably report on their own health between the ages of 4 and 6 years [49–53].

The issue of age within the context of DMD is of particular importance. With the recognised deterioration of individual’s physical ability, it could be hypothesised that QoL would deteriorate with increasing age; however, this was not confirmed by the literature. There were inconsistent findings with regards to the patients deterioration in QoL over time with some reporting changes in at least one domain according to ambulatory status [54] and others suggesting that QoL remained stable despite declines in physical functioning [24, 44, 55]. However these inferences are often based on the comparison of DMD patients in cross-sectional or case-control study designs and therefore suitably powered follow-up studies using tools which are appropriate to capture meaningful changes to QoL with age are required. It is difficult to draw conclusions regarding the effect of age on DMD based upon evidence in the literature. Future efforts to study QoL in DMD or develop a DMD-specific QoL instrument should anticipate and accommodate the complexities of eliciting responses from young children, adolescents and young adults whose ability to communication will reduce over time.

### Culture and context

Due to the inclusion of studies from around the world there were some geographical differences observed in the DMD research populations. Unsurprisingly the type and amount of formal care offered to patients and their families varies between countries [56]. One study documents that adults with DMD had higher HRQoL scores in Western Europe than those in countries in Eastern European countries [28]. One study conducted in India found that the caregivers participating in the study were mostly male, owing perhaps in part, to cultural differences in research participation [33].

### Limitations of this review

Limiting study eligibility to the last 5 years means that relevant studies published prior to 2010 may have been missed. However, as QoL themes that are known to be relevant are most likely to continue to receive attention it is less likely that important themes have not be captured by the data conducted in the last 5 years. Moreover, it could be argued that themes that have not been investigated in the last 5 years are less likely to be relevant to patients today. Similarly, the restriction to academic papers published in the English language could equally result in missed relevant themes and therefore a more inclusive approach to future comprehensive reviews is recommended. Moreover, it is also unlikely that the omission of studies will affect the output of this review as the focus is on thematic analysis. The aim is therefore to encompass the most relevant themes rather than including all published evidence. The consensus of themes discussed in the primary studies adds confidence to the probability that data saturation was reached in this review and the majority of relevant themes were captured.

### Recommendations for future research

This research highlights that factors relevant to QoL in DMD span all domains of the included HRQoL instruments and beyond. Many of the nuances specific to DMD, such as the impact on the carer and wider family, are not currently captured by standard HRQoL instruments. The breadth and diversity of themes and emerging trends in this review highlight the need to consult with patients and their families about what aspects of QoL need to be covered by the QoL instruments used. For example, themes relating specifically to accessibility and adaptation to key stages in the condition, communication, access to appropriate healthcare services, family resources, carer burden, and impact on the wider family require consideration to obtain a more holistic picture of QoL.

Health professionals and clinicians who work in DMD may be aware of the complex picture of QoL for people living with DMD but must work with researchers to raise the issue of the difficulties in capturing QoL in DMD as in other diseases occurring in children which are severe and incurable, and therefore impacting substantially on patients and carer’s QoL. Future research in this area should therefore build on the themes identified from this review and consult with people with DMD and their families. A qualitative research approach could focus on specific areas, from the variety of themes identified, that require attention and identify which should be given more weight to capture QoL in this population. This work either could inform the use of generic HRQoL instruments or could lead to the development of a specific HRQoL instrument for DMD. The aim would be to ensure that primary research conducted in the field of DMD would eventually use specific standardised, user-validated methods to study QoL.

The importance of perspective (patient versus carer) when collecting QoL data needs to be highlighted in future research. Owing to the specific nature of DMD where communication of patients’ QoL is/becomes difficult, the value of using accessible instruments that reflect user needs should be considered. Moreover, the complexities at play when interpreting differences between self and/or parent proxy report should be given due consideration.

Evidence from the literature demonstrates that DMD impacts upon an individual’s QoL, however the measurement of this has been using either existing generic instruments, condition-specific measures for given aspects of QoL (such as anxiety or depression), or using clinical indicators as a proxy. There is little robust evidence exploring the impact of DMD from the patient perspective using qualitative methods. Recent approaches to instrument development in other areas have utilised qualitative data techniques to ensure high face and content validity to the items contained within a questionnaire [57–59].

## Conclusions

Many factors relevant to people living with DMD are inadequately assessed with generic preference-based QoL instruments. Synthesising evidence from primary studies that investigate issues relevant to the QoL of people with DMD and their caregivers has generated a rich and varied profile of themes that are relevant to QoL, many of which do not easily fall under the common domains of standard QoL tools. Themes relating to phases of decline during the disease, the importance of accessible services and family resources, and the impact on the family are key to the measurement of QoL in this unique condition. Those interested in obtaining more valid estimates of QoL in this specific population should use this information to inform further qualitative research and consider which aspects of QoL are appropriate to understand and measure in DMD.

## Additional file

Additional file 1: Sample search for Embase (Ovid). (DOCX 14 kb)

## Abbreviations

BMD: Becker Muscular Dystrophy
DMD: Duchenne Muscular Dystrophy
HRQoL: Health-Related Quality of Life
LGMD: Limb Girdle Muscular Dystrophy
QoL: Quality of Life
