# Supplementary material for: A review of quality of life themes in Duchenne muscular dystrophy for patients and carers
Source: Health Qual Life Outcomes. 2018 Dec 19;16:237. doi: 10.1186/s12955-018-1062-0 (PMC6299926; doi:10.1186/s12955-018-1062-0)
Supplement: Supplementary file 1 — Sample search for Embase (Ovid). (DOCX 14 kb) [file 12955_2018_1062_MOESM1_ESM.docx]

**Additional file 1**

Sample search for Embase (Ovid)

Database: Embase <1974 to 2016 November 01>

Search Strategy:

--------------------------------------------------------------------------------

1 Duchenne muscular dystrophy/

2 duchenne*.mp.

3 1 or 2

4 health care survey/

5 exp questionnaire/ (576123)

6 (survey* or questionnaire*).mp. (1755261)

7 4 or 5 or 6 (1755261)

8 3 and 7 (732)

9 randomized controlled trial.pt. or randomized.mp. or placebo.mp. (1030748)

10 3 and 9 (571)

11 health related quality of life.tw. (42095)

12 health related qol.tw. (1759)

13 health related ql.tw. (8)

14 hrqol.tw. (16842)

15 hql.tw. (123)

16 health state utilit$.tw. (740)

17 hsuv$.tw. (71)

18 (euroqol or euro qol or eq5d or eq 5d).tw. (11588)

19 (sf6d or sf 6d or sf 6 dimension$ or sf six dimension$ or shortform 6d or shortform six dimension$ or short form 6d or short form 6 dimension$ or short form six dimension$).tw. (1076)

20 (sf12 or sf 12 or short form 12 or shortform 12 or sf twelve or sftwelve or shortform twelve or short form twelve).tw. (6383)

21 (sf36 or sf 36 or short form 36 or shortform 36 or sf thirtysix or sf thirty six or shortform thirtysix or shortform thirty six or short form thirtysix or short form thirty six).tw. (31104)

22 (item adj3 short form).tw. (5445)

23 (item adj3 shortform).tw. (10)

24 medical outcomes survey.tw. (322)

25 medical outcomes study.tw. (4461)

26 mos.tw. (11496)

27 psychological general wellbeing index.tw. (15)

28 psychological general well being index.tw. (267)

29 pgwb$.tw. (361)

30 health utilit$.tw. (2252)

31 hui$.tw. (4442)

32 quality of wellbeing.tw.

33 quality of well being.tw.

34 qwb$.tw.

35 rosser.tw.

36 trade off$.tw.

37 standard gamble$.tw.

38 tto$.tw.

39 qaly$.tw.

40 quality adjusted life year$.tw.

41 quality adjusted life year/

42 hye$.tw.

43 health$ year$ equivalent$.tw.

44 disutilit$.tw.

45 disbenefit$.tw.

46 "Quality of Life"/

47 Outcome Assessment/

48 quality of life.tw.

49 46 or 47 or 48

50 (preference based or utilit$ or generic preference).tw.

51 49 and 50

52 (preference$ adj2 (elicit$ or patient$ or population$ or measure$ or based or cost$)).tw.

53 (utilit$ adj2 (elicit$ or patient$ or population$ or measure$ or based or cost$)).tw.

54 or/11-45

55 51 or 52 or 53 or 54

56 3 and 55

57 *attitude to health/

58 *self care/

59 (patient* adj4 (feeling* or emotion* or view* or symptom* or perception* or attribute*)).ti,ab.

60 ("health related quality of life" or "health related qol" or "health related ql" or hrqol or hql or "patient reported outcome*" or "patient-reported outcome*" or prom or proms or "disease reported outcome*").ti,ab.

61 ("quality of life" or "qol" or "outcome measure*" or "health outcome*").ti,ab.

62 *"quality of life"/

63 *outcome assessment/

64 57 or 58 or 59 or 60 or 61 or 62 or 63

65 (qualitative* or findings or interview*).mp.

66 exp interview/

67 exp qualitative research/

68 65 or 66 or 67

69 3 and 64

70 68 and 69

71 8 or 10 or 56 or 70
